# Estimation of the Prevalence of Uncorrected Refractive Error and Other Ocular Morbid Conditions in School Children of Industrial Area in a Non-metro City in India

**DOI:** 10.7759/cureus.27972

**Published:** 2022-08-13

**Authors:** Prachi N Bakare, Parikshit Gogate, Renu Magdum, Supriya Phadke, Rupali Maheshgauri

**Affiliations:** 1 Ophthalmology, Pimpri Chinchwad Municipal Corporation’s Postgraduate Institute Yashwantrao Chavan Memorial Hospital, Pune, IND; 2 Ophthalmology, Dr. Gogate’s Eye Clinic, Pune, IND; 3 Ophthalmology, Community Eye Care Foundation, Pune, IND; 4 Ophthalmology, Dr. D. Y. Patil Medical College, Hospital & Research Centre, Pune, IND

**Keywords:** blindness, vision screening for school children, school eye health, prevalence of astigmatism, ocular morbidity, school children, uncorrected refractive error

## Abstract

Purpose

This study aims to estimate the prevalence of uncorrected refractive error and ocular morbid conditions in school-going children of the Pimpri Chinchwad Municipal Corporation (PCMC) industrial belt.

Methods

Ocular examination was done in a well-equipped mobile clinic on school premises in the presence of a school teacher using visual acuity (VA) charts, autorefractometer, retinoscope, and handheld slit lamp. For the age group of 5-6 years, Lea symbols and HOTV charts were used, and for the age group of >7 years, Snellen’s chart was used. A detailed anterior segment examination was done to see lid position, the presence of any lid swelling, conjunctival congestion, conjunctival xerosis, corneal opacity, and lens opacity, and findings of previous eye surgery were noted. Spectacle correction was given to these students if they were found to have a significant refractive error.

Children requiring intervention other than refractive correction were referred to a tertiary hospital.

Results

A total of 3,054 school children were examined. Most were between the age group of 11-15 years (2,448 (80.2%)), with a mean age of 12.45 ± 2.022 years; 1,470 (48.1%) were male children. A total of 368 (12.04%) children had uncorrected refractive error. Myopia was seen in 204 (6.68%) children, hypermetropia in 16 (0.52%) children, and astigmatism in 148 (4.85%) children. On classification, simple myopic astigmatism (SMA) was found in 73 (2.39%) children, compound myopic astigmatism (CMA) in 38 (1.24%) children, simple hypermetropic astigmatism (SHA) in 13 (0.34%) children, and compound hypermetropic astigmatism (CHA) in 16 (0.52%) children. Moreover, 121 children had ocular morbid conditions. Ocular morbidity with decreased vision was seen in 52 (1.7%) children with preexisting refractive error and 12 (0.39%) with amblyopia, and strabismus was seen in eight (0.26%) children. Five (0.16%) children had lens disorder, and five (0.16%) had no improvement with glasses despite normal anterior segment.

Conclusion

There was a high prevalence of uncorrected refractive error. Early detection of uncorrected refractive error and ocular morbidity will improve overall performance in school-going children.

## Introduction

Childhood blindness is a grave concern not only in India but also all over the world. Loss of vision results in a profound deleterious effect on the psychological and socioeconomic growth of not only the child but also his/her family. Moreover, the disability-adjusted life year (DALY) loss in a blind child is far greater compared to a blind adult. Therefore, control of childhood blindness was placed as a priority target in the Vision 2020: The Right to Sight initiative.

Blindness among children in India is estimated at 0.8/1,000 children [1,2].

School screenings offer a good opportunity to pick up refractive errors early and correct them in time, thereby preventing irreversible amblyopia. It also enables diagnosing and treating other ophthalmic problems in children.

The Pimpri Chinchwad Municipal Corporation (PCMC) area forms the industrial belt of Maharashtra. The urban cluster has rapidly developed in the past two decades where most of the population consists of migrants from other parts of Maharashtra and India who have settled there to work in the factories. It has an average literacy rate of 87.19%, higher than the national average of 74.04%. In this area, 14% of the population is under six years of age as given by the census 2011.

This study was carried out in municipal schools in the Pimpri Chinchwad Municipal Corporation area of Western Maharashtra, and 3,054 students between the ages of five and 18 years were screened for the presence of refractive errors and other ocular problems and prescribed glasses.

## Materials and methods

This prospective interventional study was jointly conducted by the ophthalmology department of Dr. D. Y. Patil Medical College, Hospital & Research Centre, Pune, and Community Eye Care Foundation, Pune, between December 2018 and June 2019. A total of 3,054 school children between the ages of five and 18 years from municipal schools in the suburbs of Pimpri, Chinchwad, Nigdi, Akurdi, Ravet, Bhosari, Pimple Gurav, Moshi, Punawale, and Sangavi were examined. Permission from respective authorities of PCMC (secondary school in charge and the Municipal Commissioner’s office) was taken prior to the start of the study. The Institutional Ethics Committee (research protocol number: IESC/FP/2018/13) gave permission to conduct this study.

Informed consent was taken from respective school principals. Oral consent was taken from the child and the child’s parents. In need of instillation of cycloplegic eye drops, written consent was taken from the parents.

The school vision screening team consisted of an ophthalmologist, an optometrist, an ophthalmic assistant, and a social worker. Ocular examination was done in a well-equipped mobile clinic on school premises in the presence of a school teacher.

Relevant demographic data were collected from the school authority. A detailed ocular history was taken by the optometrist. This mobile clinic was well equipped with a chair unit with vision charts, an autorefractometer, a handheld slit lamp, a self-illuminating retinoscope, and a direct ophthalmoscope.

For students in the age group of 5-6 years, the vision of each eye was checked using Lea symbols and HOTV charts and as per the recommendations for preschool vision screening program by the American Academy of Paediatrics and the American Academy of Ophthalmology. If vision in one eye or both eyes was found to be reduced to lesser than Snellen’s equivalent of 6/15 or worse in either eye or a difference of two lines or more between the eyes, retinoscopy was done with fogging method to evaluate the refractive error. In uncooperative children and children with phoria/tropia, wet retinoscopy was done after instilling cyclopentolate eye drops.

For children older than seven years of age, visual acuity (VA) assessment was performed using Snellen’s chart; pinhole vision was taken to differentiate between refractive error and ocular pathology. If the uncorrected vision was found to be less than 6/12 (Snellen) in either eye, retinoscopy was done with fogging technique, followed by subjective testing. Spectacle correction was given to these children if they were found to have a significant refractive error requiring correction, which was defined as myopia more than or equal to 0.50 D or hyperopia more than or equal to +1.00 D, and astigmatism greater than 0.50 D [3,4]. These children were dispensed spectacles free of cost. Any preexisting refractive errors were also noted.

Data regarding the pattern of refractive error were collected for analysis. Simple myopic astigmatism (SMA) was defined as myopic refractive error in one meridian and emmetropia in another. Simple hypermetropic astigmatism (SHA) was defined as hypermetropic refractive in only one meridian and emmetropic in another meridian. Compound myopic astigmatism (CMA) was defined as myopia in all meridians of differing amounts. Compound hypermetropic astigmatism (CHA) was defined as hypermetropia in all meridians of differing amounts [5]. Mixed astigmatism was defined as one meridian hypermetropic and the other myopic.

Spherical equivalence was calculated using the following formula: spherical equivalence = spherical value + (cylindrical value / 2) (in diopters) [4,6].

Further ocular examination was done using a torch and slit lamp. Visual alignment was checked using the Hirschberg test. A cover test and alternate cover-uncover test were done to rule out the presence of phorias and tropias. Prism bar cover test was performed on children with Hirschberg test showing more than 10° deviation. Extraocular movements were checked in all six cardinal positions of gaze. Ishihara charts were used to check for color vision defects.

A detailed anterior segment examination was done to see lid position, the presence of any lid swelling, conjunctival congestion, conjunctival xerosis, corneal opacity, lens opacity, and findings of previous eye surgery.

Children requiring further evaluation were sent to the eye OPD of the medical college. The results were reported to the respective parents.

The collected data were entered on a Microsoft Excel sheet (Microsoft Corp., Redmond, WA, USA) and assessed using EpiInfo7. Qualitative data were summarized using percentages, and quantitative data were evaluated using means, medians, and standard deviations. Appropriate tests of statistical significance such as a chi-squared test were used.

## Results

A total of 3,054 children were included and screened for eye and vision problems. The majority of students screened were between the age group of 11 and 15 years (2,448 (80.2%)) (Table 1), and 1,470 (48.1%) were male (Table 2). Without any refractive correction, worst eye vision of no PL was seen in one child, vision of less than 6/60 was seen in 48 children, and better eye vision of less than 6/60 was seen in 28 children, which was due to refractive error; 525 (17.1%) students had better eye vision between 6/36 and 6/12 (Tables 3, 4). On calculating spherical equivalence, refraction of less than -3.00 was seen in the right eyes (REs) of 17 (0.56%) children, while in the LEs, it was seen in 15 (0.54%) children (Table 5). A total of 368 (12.04%) children had uncorrected refractive error (Table 6). Refractive error in only one eye was seen in 133 (4.4%) children. Myopia was seen in 204 (6.68%) children, hypermetropia in 16 (0.52%) children, and astigmatism in 148 (4.85%) children. On classification, SMA was found in 73 (2.39%) children, CMA in 38 (1.24%) children, SHA in 13 (0.34%) children, and CHA in 16 (0.52%) children. Overall, 121 children had ocular morbid conditions (Table 7). Ocular morbidity with decreased vision was seen in 52 (1.7%) children with preexisting refractive error and 12 (0.39%) with amblyopia, and strabismus was seen in eight (0.26%) children (Table 8). Five children had lens disorder (0.16%), and five (0.16%) children had no improvement with glasses despite normal anterior segment.

**Table 1:** Classification as per age group

Age group (years)	Number of students	Percentage (%)
≤5	3	0.1
6-10	283	9.3
11-15	2,448	80.2
16-<18	320	10.4
Total	3,054	100

**Table 2:** Classification as per gender

Gender	Number of students	Percentage (%)
Male	1,470	48.1
Female	1,584	51.9
Total	3,054	100

**Table 3:** Better eye vision

Better eye	Number of students	Percentage (%)
3/60	2	0.1
5/60	2	0.1
6/12	174	5.7
6/18	78	2.6
6/24	43	1.4
6/36	29	0.9
6/6	134	4.4
6/60	24	0.8
6/9	2,568	84.1
Total	3,054	100

**Table 4:** Worst eye vision

Worse eye	Number of students	Percentage (%)
3/60	2	0.1
4/60	1	0
5/60	2	0.1
6/12	189	6.2
6/18	105	3.4
6/24	66	2.2
6/36	46	1.5
6/6	120	3.9
6/60	39	1.2
6/9	2,482	81.3
CFCF	3	0.1
No PL	1	0
Total	3,054	100

**Table 5:** Refraction on spherical equivalence

Refraction	Number of patients			
	Right eye	Percentage (%)	Left eye	Percentage (%)
≥-3	17	0.56	15	0.49
-1 to ≤-2.99	128	4.19	105	3.44
Up to	121	3.96	122	3.99
0	2,745	89.88	2,769	90.67
Up to 1.00	22	0.72	25	0.82
1.00-2.99	16	0.52	16	0.52
≥3.00	2	0.07	4	0.13
Total	3,054	100	3,054	100

**Table 6:** Types of refraction

	One eye		Both eyes		Total	
	Number	Percentage (%)	Number	Percentage (%)	Number	Percentage (%)
Myopia	57	1.9	147	4.8	204	6.68
SMA	28	0.9	45	1.5	73	2.39
CMA	21	0.7	17	0.6	38	1.24
Hypermetropia	8	0.3	8	0.3	16	0.52
SHA	9	0.3	4	0.1	13	0.43
CHA	7	0.2	9	0.3	16	0.52
Mixed astigmatism	3	0.1	5	0.2	8	0.26
Total	133	4.4	235	7.8	368	12.04

**Table 7:** Ocular morbidity group

Ocular morbidities	Number of students	Percentage (%)
Present	121	4
Absent	2,933	96
Total	3,054	100

**Table 8:** Prevalence of ocular morbid conditions

Ocular morbid condition	Number of students	Percentage (%)
Preexisting refractive error	52	1.7
Change in refractive error	10	0.32
Amblyopia	12	0.39
Color vision deficiency	8	0.26
Tropia	4	0.13
Phoria	4	0.13
Nystagmus	1	0.03
Microphthalmos	1	0.03
S/P enucleation	1	0.03
Lid disorders		
Ptosis	2	0.06
External hordeolum	2	0.06
Conjunctival disorders		
Allergic conjunctivitis	5	0.16
Conjunctival nevus	1	0.03
Bitot’s spot	5	0.16
Corneal disorders		
Corneal opacity	2	0.06
S/P corneal tear	1	0.03
Lens disorder		
Lens opacity (cataract)	4	0.13
Pseudophakia	1	0.03
No improvement with refraction	5	0.16
Prevalence of ocular morbidity	121	3.96
No ocular morbidity	2,933	96.04
Total	3,054	100

## Discussion

Studies on school screening for the prevalence of refractive error and ocular morbid conditions are essential in charting out specific preventive programs based on target areas through the National Programme for Control of Blindness (NPCB) [7].

The majority of students included in the study were between the age groups of 11 and 15 years. This could be due to different timings for the accommodation of primary and secondary schools on the same premises. Four students had low vision, according to the NPCB definition, in which one child had a developmental cataract, one status post (S/P) corneal tear repair, and one traumatic lens injury. The NPCB definition is based on presenting VA with correction instead of best-corrected VA. Thus, correcting refractive errors at the school level will reduce the burden of avoidable blindness [8].

A total of 368 (12.04%) children had uncorrected refractive error. Refractive error in only one eye was seen in 133 (4.4%) children. Considering good vision in another eye, refractive correction in one eye is often delayed, resulting in asthenopic symptoms. The prevalence in refractive studies was higher at 12.04% as compared to the study done by Agrawal et al. [9].

Shrestha et al. found a 10% prevalence comparable to our study [10], considering the inclusion of the same age group in both studies.

Myopia was seen in 204 (6.68%) children, and simple myopic astigmatism was seen in 73 (2.39%) children. Over four decades, the prevalence of myopia in India is 7.5% in children aged between five and 15 years as found by Agarwal et al. [11]. Hypermetropia was seen in 16 (0.52%) children. Our results were comparable to those of Krishnan et al. who reported the prevalence of myopia and hyperopia at 6.81% and 0.61%, respectively [12].

Astigmatic refractive error was seen in 148 (4.85%) children. On classification, we found SMA in 73 (2.39%) children, CMA in 38 (1.24%) children, SHA in 13 (0.34%) children, and CHA in 16 (0.52%) children. Astigmatic refractive error prevalence was noncomparable with previously published studies, where Padhye et al. found astigmatism prevalence at 0.37% [4]. However, a similar study conducted by Hashia et al. reported the prevalence of astigmatism to be 6.38% [13]. Cycloplegic retinoscopy and the availability of an autorefractometer aided in the better calculation of astigmatic refractive error among the children.

Ocular morbidity other than refractive error was seen in 4%, and uncorrected refractive error was seen in 12.04%. Amblyopia was noted among 12 (0.39%) children. Similar findings were observed by Singh et al. [14]. Squinting was seen in eight (0.26%) children, which was comparable with the studies conducted by Singh et al. (0.27%) [14] and Desai et al. (0.21%) [15].

Shrestha et al. (3.5%) [10] and Gupta et al. (2.5%) [16] noted a higher prevalence of strabismus in their studies.

Eyelid disorders were seen in 0.12%, while Agrawal et al. [9] reported 1.6% and Bigyabati et al. [17] reported 0.4%. Conjunctival disorders were seen in 0.51%, which was comparable with studies conducted in the same area by Gupta et al. [16].

Lens opacity was seen in four children. One child had a dense cataract, and the child was referred for cataract extraction. The rest needed no surgical intervention. Other ocular morbid conditions such as microphthalmos required urgent referral for retinal examination. A child with S/P enucleation wearing an ill-fitted prosthesis had cosmetic issues. Five children with no improvement with glasses were referred for detailed fundus evaluation.

Strength of the study

Screening for ocular conditions on the school premises helped in the early detection of refractive error. Comprehensive eye examination of school children was possible in schools located in remote areas.

Controlling blindness in children was a priority of Vision 2020 [18,19]. Now is the time to look beyond Vision 2020. This screening will help in identifying visual disorders. The early detection of uncorrected refractive error and strabismus will prevent amblyopia and improve overall performance in school-going children. Many conditions associated with blindness lead to childhood mortality; hence, control of blindness in children is closely linked to child survival [20]. Awareness should be created among school authorities about ocular morbid conditions. This study will help in planning future strategies directed toward the early detection and management of refractive error and ocular morbid conditions in children. School screening for ocular conditions will help the community to overcome ocular morbidities by acquiring proper treatment due to timely expert opinion.

## Conclusions

In our study, we found a high prevalence of uncorrected refractive error. Myopia, followed by astigmatism, was the most common refractive error. School screening in a mobile eye clinic on school premises will help in detecting uncorrected refractive error earlier even in remotest areas. The early detection of uncorrected refractive error and ocular morbid conditions will improve overall performance in school-going children.
